# Determination of Heavy Metal Ions and Organic Pollutants in Water Samples Using Ionic Liquids and Ionic Liquid-Modified Sorbents

**DOI:** 10.1155/2019/1948965

**Published:** 2019-10-31

**Authors:** Minglei Tian, Luwei Fang, Xuemin Yan, Wei Xiao, Kyung Ho Row

**Affiliations:** ^1^College of Chemistry and Environmental Engineering, Yangtze University, Jingzhou, China; ^2^Department of Chemistry and Chemical Engineering, Inha University, Incheon 402751, Republic of Korea

## Abstract

Water pollution, especially by inorganic and organic substances, is considered as a critical problem worldwide. Several governmental agencies are listing an increasing number of compounds as serious problems in water because of their toxicity, bioaccumulation, and persistence. In recent decades, there has been considerable research on developing analytical methods of heavy metal ions and organic pollutants from water. Ionic liquids, as the environment-friendly solvents, have been applied in the analytical process owing to their unique physicochemical properties. This review summarizes the applications of ionic liquids in the determination of heavy metal ions and organic pollutants in water samples. In addition, some sorbents that were modified physically or chemically by ionic liquids were applied in the adsorption of pollutants. According to the results in all references, the application of new designed ionic liquids and related sorbents is expected to increase in the future

## 1. Introduction

From the industrial revolution to today, environmental pollutions caused by heavy metals and toxic organic compounds are an enormous problem worldwide [1]. The pollution of rivers and streams with chemical contaminants has become one of the most critical environmental problems of the century. Heavy metals are important chemical pollutants because of their persistence in the environment [2]. The European Union Water Framework Directive set a list of priority substances that includes 33 organic and inorganic compounds that have become a serious problem in the aquatic environment because of their toxicity, bioaccumulation, and persistence.

For example, lead (Pb) is a heavy metal found widely in nature that was also commonly used for several centuries. The development of the civilization and related products increased the amount of lead emissions and caused an obvious increase in concentration in the environment [3, 4]. However, Pb is not biodegradable nor does it disintegrate in the environment. Therefore, it accumulates in the tissues of living organisms and has a high health risk, especially for children [5, 6]. Aluminum (Al) and silver (Ag) are two of the most commonly used metals in human lives due to their presence in food packing, tableware, and coins. Both can enter the environment from industrial wastewater and in recent years, there has been increasing interest in their toxicity and biological effects. Al has been implicated in encephalopathy, Parkinson's disease, and Alzheimer's disease [7], and excess amounts of Ag in the human body cause skin diseases and blood disorders [8].

Wastewater released from industrial process contains many heavy metals. Cadmium (Cd) is a nonessential element and a health hazard, even at very low concentrations in water [9]. Cobalt (Co) can be used to treat anemia, but an excess of it in the human body is harmful to the hematological systems and skin allergies can result [10]. The ingestion of more than 30.0 *μ*g/L uranium (U) from food and drinking water is toxic to the kidneys [11–15]. The International Agency for Research on Cancer listed several heavy metals as being possibly carcinogenic to humans based on animal data [16].

In addition to heavy metal toxicity, there are other issues: (1) their high affinity for water makes them difficult to remove using conventional solvents; (2) common filtration methods cannot remove them because of the minimal structures; (3) they are unaffected by natural processes, and hence, their concentration is reduced only by dilution; and (4) biological accumulation. These features can also be found in organic pollutants. Phenols, pesticides, and endocrine disrupting compounds (EDCs) are three major types of pollutants in water samples. Phenol is an organic substance used in several industrial processes, such as the production of phenolic resin and other phenol derivative chemicals. The compound is also used as a solvent, as an antiseptic, and as an additive in disinfectants [17]. Phenolic compounds are used widely in many industrial processes, such as petroleum refineries, pharmaceutical, and chemical industries. They are highly toxic compound even at low concentrations [18], and in surface water, the US Environmental Protection Agency (EPA) set a standard of less than 1.0 *μ*g/L for these compounds [19, 20]. Pesticides are used widely in agricultural practices. Approximately 800 types of pesticides have been applied worldwide, and their residues are present in soils, waters, and foods; their intensive and abusive use is a worldwide problem [21]. For example, mefenacet is an acetanilide herbicide that is used in paddy fields to control weeds [22]. Isoprothiolane is a fungicide that is used for rice to control delphacidae [23]. Dichlorodiphenyltrichloroethane (DDT) is one of the most famous pesticides in the world, but it does not degrade in the environment and can accumulate in the fat of animals and the human body. Despite its use being banned many years ago, DDT and its metabolites can be still found in the environment [24]. The guideline values for highly toxic pesticides in drinking water from the World Health Organization was less than 1.0 *μ*g/L. EDCs are chemicals that can interfere with endocrine (or hormone) systems at very low concentrations [25]. Bisphenol A (BPA) is a representative compound of EDCs. The compound is used to produce certain plastics and epoxy resins. On the other hand, the use of BPA in baby bottles and infant formula packaging is not recommended. EDCs may cause cancerous tumors, birth defects, and other developmental disorders [26]. Therefore, to avoid adverse effects on human health, the concentration of EDCs in water should be less than 0.1 *μ*g/L [27].

However, the concentrations of all pollutants in the water sample are normally quite low. In order to detect and analyze the properties in detail, assistant techniques are involved to concentrate the pollutants from water samples and can be increased the accuracy of analysis. Although traditional techniques (evaporation, extraction, crystallization, and so on) are frequently used for treating aqueous solutions, the high operational cost or difficulty in treating wastewater limits the application of most techniques, particularly when the pollutants are dissolved in large volumes of solutions. Despite the considerable improvements in modern instrumental analysis of heavy metal ions and organic pollutants, their detection is still difficult because of the low levels in samples and the high complexity of sample matrices. Although some common organic solvents were associated, the toxicity and low selectivity are one of the problems in further analysis.

In this case, a range of preconcentration processes can be used and combined with several extraction and separation methods which include liquid-liquid extraction (LLE) [28], solid phase extraction (SPE) [29–31], cloud point extraction (CPE) [32, 33], ultrasonic extraction (UE), Soxhlet extraction (SE), liquid membrane [34, 35], sorbent adsorption [36, 37], solid-phase microextraction (SPME), homogeneous liquid-liquid microextraction (LLME), single-drop microextraction (SDME), hollow fiber liquid-phase microextraction, dispersive liquid-liquid microextraction (DLLME), membrane extraction, and cold-induced aggregation microextraction (CIAME) [38–41]. The main advantages of the above-mentioned methods are their high speed and small volume of inexpensive solvent [42, 43]. Unfortunately, the disadvantages of these methods (time-consuming, unsatisfactory enrichment factors, laborious, difficult operation, and low recovery) limit their applications. In particular, the presence of large amounts of hazardous and volatile organic solvents should be avoided [44]. In this case, introducing an environment-friendly media with high extraction and separation performance is another urgent problem of analytical techniques which needs to be solved.

Recently, ionic liquids (IL), which are recognized as green solvents, have been used in efficient extraction or sorbent modification. This type of solvents are salts and liquids over a wide temperature range including room temperature and are prepared by the combination of organic cations with various anions. IL has some unique physicochemical properties, such as negligible vapor pressure, wide chemical and electrochemical windows, nonflammability, tunable viscosity and miscibility with water and organic solvents, as well as good extractability for a range of organic compounds, which make them potential replacements for organic solvents in the several areas [45–48]. Some reviews summarized several IL and their applications in analytical techniques. A review by Trujillo-Rodríguez focused on application of IL in different extraction and separation methods [49]. Another review from Clark focused on the applications of IL and IL-modified sorbents in sample preparation on extraction and biological applications [50]. Nawała et al. concluded the application of IL with the SPME method [51]. The above literatures confirmed that IL is useful and important from the detective point of view.

In this case, IL as a unique solvent can solve the problems in association with analytical techniques. First, as an environment-friendly solvent [49, 51], IL shows an excellent solubility that can extract the pollutants from a real sample even under very low concentration. Second, according to the type and structure of pollutants, IL can be designed to possess varied properties such as strength of cation/anion charge and structure or polarity of functional groups. The selectivity of designed IL will be increased apparently. Furthermore, in analytical process, several interactions (ionic bonds, H-bonds, *π*-*π* bonds, and so on) simultaneously affect the IL and pollutants. As a result, the efficiency and accuracy of analytical techniques can be extremely increased.

A large amount of research work applied IL on the analysis of pollutants. Hence, we provide an overview of recent applications of IL and IL-modified sorbents in determination of heavy metal ions and organic pollutants in various water samples.

## 2. Applications of Ionic Liquids as Solvents

Before the analysis of heavy metal ions and organic pollutants, pretreatment or concentration of water samples can efficiently increase the precision, sensitivity, and limit of detection. Liquid extraction and its derivative methods are the typical assistant techniques used in pretreatment process. Since the first use of an IL as an alternative to traditional volatile organic solvents for two phase liquid-liquid extraction, many ionic liquids have shown several advantages (solvent power, viscosity, possibility to adjust the solubility by the choice of the anion and cation to improve and the transport properties, provide ion exchange interactions, electrostatic interactions and *π*-*π* interactions, and so on) over common solvents used in separation and extraction processes [52]. Several studies have used IL as an alternative solvent to concentrate metal ions [53]. The mechanism is related to the properties of the IL, which are influenced by the charge distribution on the cations/anions, H-bonding ability, polarity, and dispersive interactions. Furthermore, their good extractability for various organic compounds and metal ions as neutral or charged complexes, as well as their tunable viscosity and miscibility with water and organic solvents, can be selected by choosing the cationic or anionic constituent [54]. For example, imidazolium IL is a highly ordered hydrogen-bonded solvent and has significant effects because of the immiscibility with water. Moreover, IL containing bis(trifluoromethylsulfonyl)imide anions ([Tf_2_N]^−^) are preferred as solvents for forming biphasic systems.  Table 1 lists all the related abbreviations and full names of the ionic liquids in this review. Some special ionic liquids were produced as “designer solvents” according to the target ions. For example, tricaprylmethylammonium chloride (Aliquat 336) was used to analyze molybdenum and wolfram, and tetradecyltrihexylphosphonium chloride (Cyphos IL 101) selectively analyzed cobalt in a mixture [55, 56].

### 2.1. Determination of Heavy Metal Ions

In traditional liquid extraction, the solvent is a hydrophobic phase compared with an aqueous metal ion solution. The hydration environment of the metal ion needs to be changed either using organic ligands that provide a more hydrophobic region around the metal, or with inorganic anions that form softer, more extractable anionic complexes with the metal [57, 58]. When IL are used as the extraction solvents, they disperse completely into the aqueous solution, and the metal ions will migrate more easily to the ionic liquid phase [59].  Table 2 provides some detail studies on heavy metal ions.

#### 2.1.1. Single Ion with Ionic Liquids

Cadmium is a nonessential element that has no positive nutritional or biometallic role in humans or animals [60, 61]. The contamination of water by Cd^2+^ is a great concern because of its ecological and occupational health hazardous effects, even at very low concentrations. Prolonged consumption of drinking water containing Cd^2+^ at levels higher than 3.0 *μ*g/L can have deleterious effects on a range of organs in the human body.

Khan et al. [62] detected Cd^2+^ using DLLME. 1-(2-pyridylazo)-2-naphthol (PAN) as a ligand was composited with Cd^2+^, and IL [BMIM][PF_6_] was used to extract the ion in water samples and human hair. The extraction behavior was then examined under a range of conditions. The pH and IL volume were two of the major conditions on the extract efficiency. pH plays an idiosyncratic role on metal-chelate formation and subsequent extraction. In this research, to achieve the highest recovery, the pH was set to 8.0. In addition, to obtain a better enrichment factor with the performance ratio of IL, extraction condition of Cd^2+^ using [BMIM][PF_6_] was optimized in methanol. The application of [BMIM][PF_6_] in water samples did not decrease the extraction efficiency. In the present study, the recovery of Cd^2+^ ions could be affected by different interfering ions. The results showed that all recoveries of Cd^2+^ were in the range of 89.3%–98.3%. Although the authors did not explain the reason, these high recoveries were probably due to the selectivity of IL.

Although chromium is an essential material for humans and plays an important role in the physiological metabolism, Cr^4+^ is a very toxic ion to humans and living organisms [63]. The maximum permissible level of Cr^4+^ in wastewater is less than 0.05 mg/L [64]. Majidi and Shemirani [65] used LLME with [BMIM][BF_4_] to concentrate Cr^4+^ ion complexed with 1,5-diphenylcarbazide (DPC) in a sulfuric acid medium. The amount of IL is one of the major factors in this study on the efficiency and enrichment factor. The extraction efficiency of Cr^4+^ obviously increased with increasing amount of IL, while the enrichment factor decreased considerably. Considering these two respects, [BMIM][BF_4_] was found to be appropriate with an enrichment factor of 100 and an extraction efficiency of 96.0% for Cr^4+^. The effect of foreign ions on the separation and determination of Cr^4+^ were tested, and under the selected conditions in that study, these ions had no obvious influence. Finally, the method was applied to detect Cr^4+^ from mineral water, sea water, and river water, and less than 3.3 × 10^−3^ *μ*g/L was detected.

Cobalt and its compounds are used in the metallurgical industry, electroplating, nuclear technology, fertilizers, medicine, and colored pigments. On the other hand, cobalt is harmful when an excess cobalt metal (more than 40.0 *μ*g per day) is taken into the human body. Hosseini et al. [41] applied *in situ* solvent formation microextraction (ISFME) to detect Co^2+^ in three water samples with [HMIM][BF_4_] as the extractant and sodium hexafluorophosphate (NaPF_6_) as an ion-pairing agent. The pH of the solution is one of the most important parameters because the pH strongly affects the formation of soluble metal complexes and their stability; a solution of pH 7.0 was suggested. Subsequently, the ion-pairing agent with different concentrations of [HMIM][BF_4_] was studied, and under the optimized conditions, foreign ions that may interfere with the determination of Co^2+^ were assessed. Because of the interactions among Co^2+^, IL, and ion-pairing agent, other ions had almost no interference with the present method. Finally, Co^2+^ in tap, lake, and rain water samples was detected with high recoveries (99.6%–101.4%). Mirzaei and Amirtaimoury [44] also detected Co^2+^ but used another two IL ([HMIM][PF_6_] and [HMIM][Tf_2_N]). Because of the common ion effect, the solubilities of these two IL decreased when they were present together. Therefore, in this study, a solution containing [HMIM][Tf_2_N] was added with small amounts of [HMIM][PF_6_] for phase separation. Other major effects such as pH and temperature were assessed and 7.5 and 0°C, respectively, were chosen. Furthermore, under optimized conditions, various amounts of interfering ions were added and they did not interfere with the determination of Co^2+^. Finally, the recoveries of Co^2+^ in mineral, tap, and river water were 92.0%–102.5%.

Lead is a heavy metal found widely in nature. The element cannot be biodegraded in the environment, so it accumulates in the tissues of living organisms and is a high health risk. The concentration of lead in wastewater should be less than 50.0 *μ*g/L. Nizamani et al. [66] performed the ultrasonic-assisted ionic liquid-based dual microextraction (UA-ILDME) of Pb^2+^ from ground and rain water with [BMIM][PF_6_] and dithizone as a chelating agent. Conditions such as pH = 6.0, amount of IL, and other effects were optimized. The effects of other metal ions were examined. Because of the selection of chelating agent, the interference on Pb^2+^ was less than 5.0%. After applying the method in water samples, 13.0–108.0 *μ*L/L of Pb^2+^ was detected.

Silver is one of the precious metals that is used widely in accessories, tableware, and coins. With its high antibacterial activity, it is used as a disinfectant in drinking water, foods, and cosmetics. Silver in such products diffuses gradually to the environment, and excess amounts in the human body cause skin diseases and blood disorders. The regulation value of silver in water has been set to 100.0 *μ*g/L by the World Health Organization (WHO). Vaezzadeh et al. [67] used modified cold-induced aggregation microextraction (M-CIAME) for the sensitive and reasonably selective determination of Ag^+^ in X-ray photographic film solutions, photographic wastewater, and lake and mineral waters. They used [HMIM][BF_4_] and 4,4-bis(dimethylamino)thiobenzophenone (TMK) as the complexing agent. Several major factors, such as pH, volume of IL, and temperature, were investigated and optimized. For example, the pH selected was 3.5 depending on the extraction yield and stability of Ag^+^. Furthermore, the effects of other metal ions were assessed. Because their TMK complexes had low stability, they had no interferences on the extraction of Ag^+^ in this study. Finally, the method was applied to detect Ag^+^ in four real samples with low RSD (1.8%) and high recoveries (high than 98.4%).

#### 2.1.2. Multiple Ions with Ionic Liquids

Aluminum, gallium, and indium are used widely in human life and industry, such as packaging materials, semiconductors, and photodetectors. The safe level of the three metal ions in drinking water is less than 0.20 mg/L, and an excess amount of these ions has been implicated in several human diseases. In this case, the three metal ions should be removed from water samples. Ghasemi and Zolfonoun [68] used UA-ILDME to detect Al^3+^, Ga^3+^, and In^3+^ in water samples (tap water, mineral water, and well water), and [HMIM][PF_6_] was selected with 1,2,5,8-tetrahydroxy anthraquinone (quinalizarine) as an ion-pairing agent. The relative recoveries for the spiked samples were in the range of 86.0–120.0%. To assess the recommended procedure of Al^3+^, Ga^3+^, and In^3+^, the effects of common coexisting ions were studied. The results showed that these ions had no effect on the determination of the three ions.

Nickel and cobalt are nutritionally essential trace metals for at least several animal species, microorganisms, and plants. Large amounts of these metals are toxic and can cause an allergic reaction in the human body. The EPA recommends that the drinking water levels should be no higher than 100.0 *μ*g/L for Ni^2+^ and 10.0 *μ*g/L for Co^2+^. Khani and Shemirani [69] used [HMIM][Tf_2_N] as a DLLME solvent with PAN as the ion-pairing agent to treat these two ions. The pH as one of the important factors affecting the formation of complexes and subsequent extraction was studied in the range of 2.0–8.0. The results showed that the absorbance of the Co^2+^ and Ni^2+^ complexes was high at pH 6.0. The effects of other 23 metal ions were evaluated. The results showed these ions had little or no effect on the determination of Co^2+^ and Ni^2+^. Finally, the proposed procedure was applied to water samples (tap and mineral water); Co^2+^ was not found, and Ni^2+^ was detected at approximately 5.0 *μ*g/L.

Yao et al. [70] used ultrasound-assisted magnetic retrieval-linked ionic liquid dispersive liquid-liquid microextraction (UA-MR-IL-DLLME) to detect Cd^2+^ and Pb^2+^ in tap, river, and well water samples with three IL ([BMIM][PF_6_], [HMIM][PF_6_] and [OMIM][PF_6_]) and ammonium pyrrolidine dithiocarbamate (APDC) as the complexing agent. According to the highest efficiency, [HMIM][PF_6_] with the lowest viscosity was selected. Then, effects of other ions on the method were determined. The recoveries of the two ions were higher than 95.3%, showing the high selectivity of the IL. Finally, the recoveries in the acceptable range of 97.1%–101.6% in the three water samples confirmed the accuracy of the method.

Werner [71] analyzed Ni^2+^, Co^2+^, Cd^2+^, Cu^2+^, and Pb^2+^ using Cyphos IL 104 in river and lake water with ionic liquid ultrasound-assisted dispersive liquid-liquid microextraction based on solidification of the aqueous phase method (IL-UA-DLLME-SAP). Under pH = 5.0, the RSDs and recoveries were in the range of 2.0–6.0% and 97.0–102.0%, respectively.

Fischer et al. [72] conducted more detail studies on the microextraction of 11 metal ions with eight synthesized IL [A336][TS], [A336][MTBA], [A336][BA], [A336][Hex], [A336][SCN], [PR4][TS], [PR4][MTBA], and [PR4][Sal] without an ion-pairing agent. For Hg^+^ and Ag^+^, depending on the high solubility in all studied IL, the extraction efficiencies for both ions were higher than 80.0%. For Pt^4+^, [A336][SCN] and [PR4][MTBA] revealed extraction efficiencies more than 95.0%. For Cu^2+^ and Sn^2+^, [A336][TS] can achieve high extraction efficiencies as 95.0% and 82.0%. For Cd^2+^, Pb^2+^, and Zn^2+^, the highest extraction efficiencies were 38.0% by [PR4][MTBA], 41.0% by [PR4][TS], and 43.0% by [A336][SCN]. For As^3+^, Cr^3+^, and Ni^2+^, the extraction efficiencies by all IL were less than 16.0%. In summary, the viscosity, functional groups, cation ring, and cation-anion affect the extraction behaviors. The method was then applied to detect ions in six wastewater samples. The results showed that the extraction efficiencies of some ions in real samples were much higher than those in the model solution. One factor is that the extent of extraction depended on the initial concentration the metal ion present in the aqueous solution. When the concentration of the ion was low in wastewater samples, IL could fully concentrate the ions in which the initial concentration was low in the wastewater sample. Another factor influencing the extraction efficiency is the pH of the system that can control the ionic strength. Therefore, for Ni^2+^, Zn^2+^, and Pb^2+^, the IL could extract more when the pH changed from 7.5 to the range of 7.9–9.4.

Several studies investigated the extraction effects of interfering metal ions that were common coexistence ions in water samples. Although they had a competitive interaction with the chelating agent, selective extraction can be adjusted using different IL interacting with functional groups on metal ion-chelating agent complex. In addition, pH is a significant factor in metal-chelate-IL formation and is a key parameter for extraction. Therefore, the selected IL can extract the target metal ions with high recoveries under the optimized pH.

### 2.2. Determination of Organic Pollutants

In the concentration and determination process of organic pollutants, based on different interactions between the receptor and dispersing solvent, the pollutants could be concentrated in different phases. Therefore, when IL was used in aqueous systems, the hydrophilic/hydrophobic properties of IL could affect the extraction efficiency. To reveal the interaction mechanism and chemical bonds, Gao et al. [73] studied the interactions between dimethyl sulfoxide (DMSO) and using 10 IL ([EMIM]Cl, [BMIM]Cl, [HMIM]Cl, [OMIM]Cl, [AMIM]Cl, [BDMIM]Cl, [EMIM]Br, [BMIM]Br, [HMIM]Br, and [OMIM]Br). A method called an aqueous biphasic system (ABS) was applied to separate the water phase and another phase because of the different properties of each component. Second, the ability of the IL to concentrate DMSO was investigated, and several chemical interactions were described. (1) The hydrogen at the IL cation formed a weak hydrogen bond (C-H⋯∙∙∙O) with the oxygen atom of DMSO, and a higher amount of DMSO formed more C-H⋯O bonds. (2) The hydrophobicity of IL increased with increasing the alkyl side chain length. The hydrophobicity decreased the hydrogen bond basicity (*β*) in ABS, so the efficiency of the IL increased. (3) The increasing of the alkyl side chain length leads to an increase in dispersive interactions among the IL ions. (4) Increasing the size of the alkyl side chain of the IL would decrease the surface tension to dissolve DMSO. (5) When the alkyl chain length in IL was larger than six, the reversed micelle formation would decrease the DMSO concentration. Finally, the effects of temperature and pH were evaluated. The hydrogen bonds weakened with increasing of temperature, so the partition coefficients of DMSO reduced at higher temperatures. Moreover, the high pH of the system can induce ABS and extract more DMSO.

From the results of previous research, the hydrophilic/hydrophobic properties and chemical bonds of IL are two major mechanisms to promote the concentration of organic compounds. On the other hand, one of the IL characteristics exploited is their ability to dissolve a variety of solutes by modifying and combining suitable cations and anions. Therefore, IL is a novel, environmentally benign solvent for the analysis of organic pollutants [74, 75]. For example, IL can be used in place of traditional organic solvents in LLE, where hydrophobic molecules, such as simple benzene derivatives, will partition to the IL phase [76, 77].  Table 3 lists some detailed studies on organic pollutants.

Phenolic compounds are common organic compounds used in many manufacturing processes and chemical solvents. On the other hand, because of the carcinogenic effect, they are toxic to humans and the environment [78]. To detect them, several ionic liquids have been applied. Balasubramanian et al. [79, 80] conducted two studies to detect five phenolic compounds (phenol, p-chlorophenol (CP), 2,4-dichlorophenol (DCP), 2,4,6-trichlorophenol (TCP), and pentachlorophenol (PCP)) using a liquid membrane fabricated by dissolving one IL in tributyl phosphate (TBP) to obtain an ionic liquid mixed carrier. First, [BMIM][PF_6_] in TBP could concentrate 99.5% of the phenols. As the IL concentration increased, the amount of phenol decreased to 98%. [BMIM][PF_6_] at high concentrations in the membrane phase promoted its insolubility but did not enhance phenol removal at very low concentrations. Second, the IL Aliquat 336 was selected. Using the same method and influence factors, 99.3% of phenols were decreased. In both studies, the effects of external phase pH were studied in the range of 2.0–10.0 because the phenols concentrating depended on hydrogen bonding (between the hydroxyl H of the phenols with the Cl^−^ anion of the ionic liquid) and hydrophobic interactions (between the cation of IL with phenols). Finally, pH = 6.5 was chosen as the optimal value. Sas et al. [81] detected phenol, o-cresol, 2-chlorophenol, and resorcinol using [BMIM][Tf_2_N], [HMIM][Tf_2_N], and [HMIM][Tf_2_N]. When the pH was lower than 6.0, the highest efficiency would be 99.99%.

Almeida et al. [82] used seven IL ([BMIM][SCN], [BMIM][TOS], [BMIM][N(CN)_2_], [BMIM][CH_3_CO_2_], [BMIM]Cl, [BMPIP]Cl, and [BMPYR]Cl) to analyze three phenolic acids using an aqueous two-phase system (ATP) method. The anion and cation effects on both IL on the ATP method were evaluated. When the cation was fixed to [BMIM]^+^, the abilities to form ATP were [CH_3_CO_2_]^−^ ≈ Cl^−^ < [TOS]^−^ < [SCN]^−^ [N(CN)_2_]^−^ because of the hydrophobicity of the anion. When the anion was Cl^−^, the low affinity of the cation for water showed higher ability to form ATP ([BMIM]^+^ < [BMPYR]^+^ < [BMPIP]^+^).

Nitroaromatic compounds are quite toxic, even at low concentrations. They can be released into the environment through waste from the chemical industry and other industries. Berton et al. [83] reported the properties of two IL [HMIM][Tf_2_N] and [C_6_MPY][Tf_2_N] on the microextraction of 4-nitrotoluene (NT), 2,6-dinitrotoluene (DNT) and 2,4,6-trinitrotoluene (TNT) in water samples. With a less viscous anion and lower solubility in water, [C_6_MPY][Tf_2_N] was selected as the accepting solvent. Software with a design process was used to assist in the experiments, and the results showed that the pH and buffer concentration could not influence the extraction efficiency because of the nonionizable property of the three compounds. Under the optimizing conditions, the method was applied in two real samples, and the average relative recoveries were higher than 93.2%.

Some studies compared the influences of different cations or anions of IL on the analysis of pesticides. DDT is a pesticide that was used all over the world several decades ago and can still be found in the environment. Wang et al. [24] developed an ionic liquids-DLLME method in which two types of IL with opposite properties (hydrophobic or hydrophilic) were used as the extraction solvent and disperser solvent, respectively. The authors applied this method to detect DDT and its metabolites (*o*,*p'*-DDT; *p*,*p'*-DDT; *p*,*p'*-DDD; and *p*,*p'*-DDE) in four water samples with six IL ([BMIM][PF_6_], [HMIM][PF_6_], [OMIM][PF_6_], [BMIM][BF_4_], [EMIM][BF_4_], and [BMIM]NO_3_). First, [BMIM][PF_6_], [HMIM][PF_6_], and [OMIM][PF_6_] were chosen. DDT and its metabolites are nonpolar or weakly polar compounds, and the polarity order of the three imidazolium-IL is as follows: [BMIM][PF_6_] > [HMIM][PF_6_] > [OMIM][PF_6_]. Therefore, [OMIM][PF_6_] was firstly used to deal with the water sample. Second, the disperser solvent was tested using three hydrophilic IL ([EMIM][BF_4_], [BMIM][BF_4_], and [BMIM]NO_3_). The experimental results showed that [BMIM][BF_4_] can achieve the highest extraction recoveries of DDT and its metabolites. Finally, in subsequent experiments, [BMIM][BF_4_] was used as a disperser solvent. In addition, pH over the range 3.0–11.0 and NaCl concentration in the aqueous solution in the range of 0.0–20.0% were investigated. The state of the extraction solvent [OMIM][PF_6_] was changed in both acidic and alkaline aqueous environments, and NaCl addition decreased the extraction efficiency. Therefore, the pH was adjusted to 7.0, and no NaCl was added. Four real environmental water samples were analyzed; no DDT or its metabolites were found. The spiked recoveries were satisfactory in the range of 85.7–106.8% with a RSD of 6.0–8.5%.

BPA is a widely used EDC in the plastic industry and can influence the generative function of humans at low concentration in the wastewater system. López-Darias et al. [84] applied the IL-DLLME method to detect BPA, 4-cumylphenol (4-CP), 4-*tert*-butylphenol (t-BP), 4-octylphenol (OP), 4-*tert*-octylphenol (t-OP), and 4-*n*-nonylphenol (NP) in sea water and industrial effluents. In this method, [BMIM]Cl as a hydrophilic IL was added to an aqueous solution of the phenols. When all phenols were dissolved in the IL, the ion-pairing reagent, LiNTf_2_, was added to form a hydrophobic IL [BMIM][Tf_2_N] that concentrated all the phenols from aqueous solution. The method was applied to real samples, and an efficiency of 12.0–13.8% and recovery of 106.0–111.0% of BPA were obtained. Jiang et al. [85] selected one IL [OMIM][PF_6_] and developed a new method combining cold-induced aggregation with microextraction (CIA-ME) to detect the BPA and 2-naphthol levels in water samples. Major factors, such as the volume of IL, pH, and temperature, were investigated. The optimized results showed that 97.1–108.1% recoveries could be obtained, and pH had no influence on analysis. Qi et al. [86] synthesized an IL TOAP and applied it to the aqueous two-phase microfluidics (ATPM) system to detect BPA in an aqueous solution. Two major conditions, such as temperature and pH, were investigated. The solubility of BPA in water increased with increasing temperature, so the system was carried out at room temperature. Furthermore, BPA is a weak acid in aqueous solution, and the highest extraction efficiency was obtained at pH = 7.0. Finally, the method under the optimized conditions was applied to detect the BPA in tap, reservoir, and beach water samples. The recoveries of standard addition for all water samples spiked with BPA ranged from 95.5 to 109.9% with the RSD of 2.9–4.5%.

17-*β*-Estradiol-benzoate, 17-*α*-estradiol, and quinestrol are also three EDCs. Zhang et al. [26] used microextraction to detect these EDCs in lake, well, tap, and river water. [EMIM][BF_4_], [BMIM][BF_4_], and [HMIM][BF_4_] were used as the extraction solvents, and [NH_4_][PF_6_] was used as an ion-pairing agent. According to the aqueous solubility of IL and chemical bonds between IL and analytes (hydrogen bonding, *π*-*π*, or *π*-n), [BMIM][BF_4_] was selected as the optimal extraction solvent.

Gao et al. [87] used salting-out-enhanced ionic liquid microextraction based on a dual-role solvent (SILM-DS) with three IL [BMIM][PF_6_], [HMIM][PF_6_], and [OMIM][PF_6_] to detect five EDCs tetracycline (TCL), doxycycline (DOX), BPA, triclosan (TCS), and methyl triclosan (MTCS) in milk and water samples. The pH in the range of 2.0–8.0 was investigated, and 2.0 was chosen from the results. The effects of the type and volume of IL were tested. Phase separation could extract the analytes from water because of the aqueous solubility of IL, so [OMIM][PF_6_] was selected. Finally, the method was applied in real samples, and the recoveries were 74.5–106.9%.

Overall, IL can detect the heavy metal ions and organic pollutants in water samples efficiently. The hydrated nature of the metal ions explains their affinities to water. Therefore, the hydration state needs to be altered using a chelating agent to form a complex and provide a more hydrophobic structure from water samples. In addition, metal ions are positively charged in aqueous solution. With designed anions, for example, eight IL with different anions in Fischer's research [78], IL has different behavior. Therefore, the complexes tended to be better solvated by the specifically selected IL, which had stronger interactions on the complexes or even directly on the metal ions [88]. Furthermore, based on different types and ratios of the IL-chelating agent system, IL can interact with designated metal ions according to ionic and other chemical bonds under a specific pH condition. Hence, IL selectively combined with the target metal ions during the determination process.

From the literature related to the organic pollutants, it was found that IL is strongly solvated by hydrogen-bonding solvents, principally by forming hydrogen bonds with the anions. On the other hand, hydrophobic interactions between the cation of IL and benzene/phenol functional group on the phenolic compounds and EDCs are also very important. Based on these results from the literature, the organic pollutants that can be bound to IL by hydrogen bonding and hydrophobic interactions are transferred easily from aqueous solution to the IL phases.

In the results of all above-mentioned techniques, the research studies using in association with the ionic liquid showed relatively low LODs, high recovery, and high efficiency. In addition, the overall precision of the current method in terms of RSD was better or comparable than other methods. Hence, the IL with the appropriate anion and cation will enhance the facility for determination.

## 3. Applications of Ionic Liquids as Sorbents Modifiers

Determination methods are necessary to evaluate the removing efficiency of heavy metal ions and organic pollutants from water samples. The most commonly used techniques for determining the amount of cadmium ions in aqueous solution include UV-visible spectrometry, atomic fluorescence spectrometry (AFS) [89], flame atomic absorption spectrometry (FAAS), electrothermal atomic absorption spectrometry (ETAAS), inductively coupled plasma mass spectrometry (ICP-MS) [90, 91], X-ray fluorescence spectrometry [92], and high-performance liquid chromatography (HPLC). These techniques provide sensitive and accurate results at low concentration of analytes because of the complexity, high capital and operational costs, and time consumability of the systems [93]. On the other hand, they have several disadvantages, such as the requirement of expensive apparatus, complicated operation, high operation cost of operation and maintenance, and the requirement of well-controlled experimental conditions [94, 95].

In this case, preconcentration methods such as the classical LLE and SPE are proposed. On the other hand, these methods require large quantities of organic solvents that are still harmful to the environment [96]. Other treatment methods such as chemical precipitation/oxidation, ion exchange, membrane processes, electrodialysis, reverse osmosis, photocatalytic degradation, coagulation, flocculation, oxidation/reduction, sedimentation, filtration, solvent extraction, and adsorption are classified as primary water treatment technologies because of their high efficiency and flexibility, low cost, separation, and simplicity of design and operation [97–99].

Among the treatments to concentrate heavy metal ions and organic pollutants, adsorption is the simplest and most dynamic physicochemical process that has been employed for the removal of a range of toxic pollutants present in water systems [100]. In this process, a suitable sorbent is needed to complete adsorption. Various substances, such as biomaterials, activated carbon [101, 102], carbon nanotubes [103], graphene oxide [104], TiO_2_ [105], metal-organic framework (MOF) [106], polymer [107], and silica gel [108], have been exploited as adsorbents.

Although IL is considered as efficient liquid extractants and can interact with metal ions or organic pollutants through hydrophobic, *π*-*π* interactions and other mechanisms [109], several drawbacks such as large consumption, difficult recycling, and tedious separation procedures have restricted their applications, for example, chelating agent as required with IL in LLE of metal ions [110, 111]. In addition, during the IL extraction, the cations or anions would be lost to the aqueous phase. The decomposition of the IL phase and pollutants to the aqueous phase limits the applications of IL to extraction [112]. Therefore, new sorbents by mixing and grinding IL with previous solid substances were carried out and both advantages were involved. The adsorption mechanism would be related to the surface area of the substances and the properties of the modified IL. With a large surface area, substances can provide more groups for adsorption and modification. In addition, IL maintains their properties when immobilized on substances. Therefore, IL-modified substances have been used in separations owing to their polarity, H-bonding, ion exchange, electrostatic interaction, and *π*-*π* interactions between the target and the IL functional groups [113–115]. According to the different target pollutants, the stability of the substances under different pH environments, type of anions, and cations with different alkyl chain lengths and additional functional groups on IL will obviously affect the adsorption efficiency.

In the preparation of sorbents, two modified methods, such as impregnation and chemical modification, were used. In the preparation of an impregnated sorbent, IL and substrate were mixed and stirred in a solvent for several hours. The operation and process were quite simple and convenient. On the other hand, the chemical bonds between the IL and substrates were mainly van der Waals force or hydrogen bonds, which were neither strong nor stable enough. Therefore, some researchers chemically immobilized IL groups on substrates by covalent bonding to increase the stability and efficiency of the sorbents.  Table 4 lists all preparation methods of IL-modified sorbents, and Table 5 lists the results of the adsorption of heavy metal ions and organic pollutants in the various sorbents.

### 3.1. Adsorption of Heavy Metal Ions

Because of the excellent stability of inorganic substrates, several studies applied these sorbents, particularly carbon-based materials and silica, to adsorb pollutants from water samples. Activated carbon, carbon nanotubes (CNT), and graphene are three of the most important carbon-based materials with a large specific surface area, and they provide high efficiency to extract various metal ions. Ismaiel et al. [95] developed an IL-modified activated carbon as an electrode component. The IL-modified sorbent was prepared by mixing the measured palm shell-activated carbon (PSAC) powder and TOMAS. The method was then applied to detect Cd^2+^ in four drinking water samples with relative standard deviations (RSD) of 1.6–3.2% and recoveries of 97.8–104.6%.

Afkhami et al. [116] developed two sorbents to detect Pb^2+^ and Hg^2+^. One sorbent was produced by the composition of ionophore, [BMIM][PF_6_], graphite powder, MWCNT, and silica. And then, the sorbent was used to detect Pb^2+^ in tap, river, and waste water samples. In the pH range of 4.5–8.0, the sorbents could adsorb Pb^2+^ with the recoveries of 95.0–102.0%. The other sorbent adsorbed Hg^2+^ in sea water and wastewater [94]. The sorbent was made by mixing graphite powder, [BMP][Tf_2_N], and MWCNT. The pH was selected as 3.0 because of the degradation of the ligand and the hydrolysis of Hg^2+^.

Rofouei et al. [117] used [BMIM][PF_6_] to modify magnetic graphene oxide (MGO). The obtained sorbent was then applied to detect six heavy metal ions (Cu^2+^, Co^2+^, Cr^2+^, Ni^2+^, Zn^2+^, and Cd^2+^) in an aqueous solution with PAN as the ion-chelating agent. The adsorption efficiency on the sorbent was optimized according to the pH and extraction time. The results showed that pH = 9.0 and 10.0 min extraction time to be optimal. Subsequently, the six ions were extracted by the sorbent in the waste, river, and mineral water samples with RSD ≤ 3.2%. Gu and Zhu prepared another IL-MGO [118]. [HMIM]Gly was used, and the sorbent was applied to extract four metal ions (Al^3+^, Cr^3+^, Cu^2+^, and Pb^2+^) from three water samples. The pH in the range of 3.0–11.0 was investigated. Al^3+^ reached adsorptive equilibrium at pH = 7.0, while the adsorption efficiencies of Cr^3+^, Cu^2+^, and Pb^2+^ reached a maximum at pH = 9.0. Moreover, the adsorption efficiencies of the four metal ions were greater than 90.0% after 5.0 min, so it was selected as the optimal adsorption time. Under the optimized condition, the adsorption capacities of the four ions on the sorbent were 5.9, 5.9, 45.0, and 11.7 mg/g.

Silica also has a large specific surface area, and a large number of –OH groups on its surface, making it easy to be modified by IL groups. Wen et al. [108] developed a [NH_2_EBIM][PF_6_]-modified silica with the SPE method to detect Cd^2+^. The RSD was 2.3%, and the recoveries were in the range of 97.0–104.0%. Mahmoud and Albishri [110] modified the surface of a nanosilica amine sorbent with two IL [EMIM][Tf_2_N] and [OMIM][Tf_2_N]. The two sorbents were then used to detect Cd^2+^ in drinking tap water and wastewater samples. At pH = 1.0, 1.2 mmol/g and 1.1 mmol/g of Cd^2+^ could be adsorbed. Mahmoud et al. [119] modified silica using [CNC_3_MIM][Tf_2_N] and detected Pb^2+^ and Cd^2+^ in three water samples. The highly adsorption efficiencies of the sorbent were shown with recoveries in the range of 97.4–99.8 at pH = 7.0.

Organic substrates have other advantages, such as various functional groups and controllable porous structure. Hence, they are also applied to prepare IL-modified sorbents. Cyphos IL 101 was already applied in metal ion extraction, and Navarro et al. [120–125] impregnated Cyphos IL 101 on resin for the adsorption of five metal ions. Based on this method, Escudero et al. [96] impregnated Cyphos IL 101 on each Amberlite XAD-4, XAD-16, and XAD-1180 polymeric resins. The Cyphos IL 101-modified XAD-1180 resin was chosen because XAD-1180 resin showed the best performance for retention of the analyte. When the material was applied to detect Hg^2+^ in three water samples by SPE, the recoveries for spiked Hg^2+^ species ranged from 96.2% to 103.0%.

### 3.2. Adsorption of Organic Pollutants

IL-modified silica sorbent was also applied to the adsorption of organic pollutants. Marwani and Bakhsh [105] prepared [ClPr][Tf_2_N]-modified silica to adsorb 4-CP in drinking, ground, lake, sea, and waste water samples. The effect of pH was investigated, and the adsorption capacity of SiO_2_-ClPrTf_2_N for 4-CP decreased with increasing pH. Therefore, pH = 1.0 was selected, and the maximum capacity on the sorbent was 626.3 mg/g. When the sorbent was applied to water samples, 88.6 to 98.1% of 4-CP could be extracted with an average RSD of 4.9%.

Considering the various groups in organic pollutants, researchers paid more attention to IL-modified organic substrates. Two IL-impregnated organic sorbents were prepared to adsorb dibutyl phthalate (DBP) and polycyclic aromatic hydrocarbons (PAH). Phthalates (PAE) are classified as EDCs and priority pollutants with dibutyl phthalate (DBP) being one of the most common PAE present in environmental samples. Qureshi et al. [100] used [BMP][Tf_2_N] to modify XAD-4 resin, and the modified sorbent was used to treat four environmental water samples. At pH = 6.0, 20.0% of DBP could be adsorbed. PAH are generally used as intermediaries in agricultural, pharmaceutical, and other chemical industries. On the other hand, some of them are priority pollutants that can cause cancer. Nasrollahpour et al. [126] extracted 12 PAH (naphthalene (Naph), anthracene (Ant), phenanthrene (Phe), pyrene (Pyr,), benzo[a]pyrene (B[a]P), benzo[a]anthracene (B[a]ANTH), benzo[b]fluoranthene (B[b]FLAN,), chrysene (CH), benzo[k]fluoranthene (B[k]FLAN), indeno[1,2,3-cd]pyrene (I[123-cd]PY), dibenzo[a,h]-anthracene (D[a,h]AN), and benzo[ghi]perylene (BghiPer)) from a water sample using IL-modified MOF. Several extraction conditions, such as pH, amount of sorbent and time, were optimized. pH = 7.0 was selected because the PAH are all nonionizable compounds in aqueous solution, so a change in pH had no effect on extraction. The sorbent was applied in mineral, river, and sea water samples, and the results showed that the recoveries were in the range of 97.0–103.5%, and the RSD were 3.3–7.2%.

Furthermore, IL were chemically modified as sorbents to increase the adsorption efficiencies. Raoov et al. [127] synthesized a cyclodextrin-ionic liquid polymer (*β*-CD -[BIM][TDI]). The sorbent was applied to detect six phenols (2-chlorophenol (2-CP), 2-nitrophenol (2-NP), DCP, 4-chlorophenol(4-CP), 4-chloro-3-methylphenol(4-CMP), and TCP) in two real water samples using the SPE method at pH = 6.0, the recovery and RSD were 87.0–116.0% and 0.1–1.7%, respectively. Zhu et al. [128] immobilized [C_4_MIM]Br on silica and applied it to detect four phenols in several wastewater samples. With the SPE method, the recovery was in the range of 71.1–115.7%, and RSD were 1.1–11.3%.

p-Nitroaniline (p-NA) is an aromatic amine, and it is important for the synthesis of chemical products. On the other hand, it may poison the blood of humans and even cause cancer. Lu et al. [129] synthesized a molecularly imprinted polymeric ionic liquid (MIPIL) microsphere to detect *p*-NA from wastewater samples. The resultant sorbent had a high selective recognition force because the electron-rich group, alkenyl imidazole, in the IL functional monomer improved the *π*-*π* stacking, electronic, and hydrogen bonding between the *p*-NA and MIPIL. When the sorbent was used in river, tap, and lake water samples, the recoveries were 89.0–114.0%.

Chlorsulfuron (CS) is a widely used herbicide that is found frequently in environmental water samples. Guo et al. [130] prepared [VBIM]Cl-modified molecularly imprinted polymer (MIP) to extract CS by SPE. Under the optimized conditions, such as pH range of 4.2–4.6, the sorbent was applied to three real water samples with the recoveries and RSDs of 81.0–110.1% and 1.2–7.6%, respectively.

Overall, the selectivity of sorbents increased with specially designed anion/cation IL. The adsorption of metal ions and organic pollutants was largely dependent on their hydrophilicity and the pH of the solution. With the exception of the properties of IL, such as H-bonding and polarity, ionic bonds had major impacts on adsorption. The IL-modified sorbents preferred to form ionic bonds between metal ions and anions on IL and between negative charge organic ions and cations on IL. On the other hand, because of the stronger ionic bonds between the metal ions and IL, the adsorption efficiency of metal ions on the sorbents was higher than that of organic pollutants. Therefore, researchers preferred to evaluate the adsorption of metal ions in water than organic pollutants. In addition, the substrates can greatly enhance the adsorption capacity because of their high specific surface area and tunable pore sizes. In the literature, the substrates were normally inorganic (such as silica and carbon) and organic (such as polymer and MOF) materials. Inorganic substrates have a huge surface area and provided large numbers of functional sites for IL modification. The structure of organic substrates can be adjusted easily by porogens or templates. Furthermore, without volatile organic solvents, the adsorption method using the IL-modified sorbent is simple, rapid, reproducible, and environmentally friendly. Also, the sensitivity is satisfactory and comparable to other reported methods.

## 4. Conclusion

When IL was used as a green solvent to detect pollutants, according to properties of the target substances, IL with different types of cations and anions were selected according to the different hydrogen bond basicity strength, viscosity, hydrophilicity, and hydrophobicity. On the other hand, heavy metal ions in water samples were hardly combined by pure IL because the hydrated ions preferred the aqueous phase, and conventional IL have no selectivity. To increase the analysis accuracy, two methods in the above studies were adopted. First, the ions could be successfully concentrated into IL-complexed ion-pairing agent with the associated methods. Second, functionalized IL that contains metal ion coordinating groups incorporated covalently into the cation or anion were designed. The hydrophilicity/hydrophobicity of IL is the main interaction in determination of organic pollutants. The methods with assistance of IL proved to be a rapid, simple, sensitive, precise, and accurate analytical approach. Although the analysis using low volumes of IL provided a simple, economic, and environmentally friendly operation process, there were still some drawbacks, such as the lower selectivity for metal ions, difficulty in phase separation, and entraining loss of ionic liquid to the aqueous phase. IL-modified sorbents can solve these problems, and in the adsorption process, the sorbents could obviously improve the adsorption capacity. Nevertheless, additional agents and low efficiency on sorbent modification limited the application of IL in the determination process. Therefore, in the future, newly designed IL may provide considerable assurance for applying them as an effective approach for determination of pollutants in complicated and variable water samples.

## Figures and Tables

**Table 1:** Full names of anion/cation and abbreviations of all ionic liquids.

Anion abbr.	Full name	Cation abbr.	(Full name)	ILs
[BA]	Benzoate	[A336]	Tricaprylmethylammonium or aliquat	[A336][BA]
[BF_4_]	Tetrafluoroborate	[MIM]	Methylimidazolium	[MIM][BF_4_]
		[C_2_MIM] or [EMIM]	1-Ethyl-3-methylimidazolium	[C_2_MIM][BF_4_]
		[C_4_MIM] or [BMIM]	1-Butyl-3-methylimidazolium	[C_4_MIM][BF_4_]
		[C_6_MIM] or [HMIM]	1-Hexyl-3-methylimidazolium	[C_6_MIM][BF_4_]
		[C_8_MIM] or [OMIM]	1-Octyl-3-methylimidazolium	[C_8_MIM][BF_4_]
Br	Bromide	[C_2_MIM], [C_4_MIM],		[C_2_MIM]Br, [C_4_MIM]Br,
		[C_6_MIM], [C_8_MIM]		[C_6_MIM]Br, [C_8_MIM]Br
[CH_3_CO_2_]	Acetate	[C_4_MIM]		[C_4_MIM][CH_3_CO_2_]
[Cl]	Chloride	[C_2_MIM], [C_4_MIM],		[C_2_MIM]Cl, [C_4_MIM]Cl,
		[C_6_MIM], [C_8_MIM]		[C_6_MIM]Cl, [C_8_MIM]Cl
		[A336]		[A336]Cl or Aliquat 336
		[AMIM]	1-Allyl-3-methylimidazolium	[AMIM]Cl
		[BDMIM]	1-Butyl-2,3-dimethylimidazolium	[BDMIM]Cl
		[C_4_MPIP] or [BMPIP]	1-Butyl-1-methylpiperidinium	[C_4_MPIP]Cl
		[C_4_MPYR] or [BMPYR]	1-Butyl-1-methylpyrrolidinium	[C_4_MPYR]Cl
		Cyphos 101	Trihexyl(tetradecyl)phosphonium	Cyphos IL 101
		Cyphos 167	Tributyl(tetradecyl)phosphonium	Cyphos IL 167
		[VBIM]	1-Vinyl-3-butylimidazolium	[VBIM]Cl
		3-(anthracen-9-ylmethyl)-1-vinyl-1H-imidazol-3-ium		—
[BnSAc]	Benzylsulfanyl acetate	[N_1888_]	Methyltrioctylammonium	[N_1888_][BnSAc], [P_1888_][BnSAc];
[C_4_SAc]	Butylsulfanyl acetate	[P_1888_]	Methyltrioctylphosphonium	[N_1888_][C_4_SAc], [P_1888_][C_4_SAc],
[C_5_SAc]	Pentylsulfanyl acetate			[N_1888_][C_5_SAc], [P_1888_][C_5_SAc],
[C_6_SAc]	Hexylsulfanyl acetate			[N_1888_][C_6_SAc], [P_1888_][C_6_SAc],
FAP	Tris(pentafluoroethyl)trifluorophosphate	[C_4_MIM]		[C_4_MIM][FAP]
Gly	Glycine	[C_6_MIM]		[C_6_MIM]Gly
[Hex]	Hexanoate	[A336]		[A336][Hex]
P	Propionate	TOA	Tri-*N*-octyl ammonium	TOAP
[MTBA]	2-(Methylthio)benzoate	[A336]		[A336][MTBA]
		[PR4]	Trihexyl(tetradecyl)phosphonium	[PR4][MTBA]
[N(CN)_2_]	Dicyanamide	[C_4_MIM]		[C_4_MIM][N(CN)_2_]
NO_3_	Nitrate	[C_4_MIM], TOMA		[C_4_MIM]NO_3_, TOMAN
[PF_6_]	Hexafluorophosphate	[C_4_MIM], [C_6_MIM],		[C_4_MIM][PF_6_], [C_6_MIM][PF_6_],
		[C_8_MIM]		[C_8_MIM][PF_6_]
		[NH_4_]	Ammonium	[NH_4_][PF_6_]
[SCN]	Thiocyanate	[A336], [C_4_MIM]		[C_4_MIM][SCN], [A336][SCN]
[SAl]	Salicylate	[PR4], TOMA		[PR4][Sal], TOMAS
[Tf_2_N]	Bis(trifluoromethylsulfonyl)imide	[C_2_MIM], [C_4_MIM],		[C_2_MIM][Tf_2_N], [C_4_MIM][Tf_2_N],
		[C_6_MIM], [C_8_MIM]		[C_6_MIM][Tf_2_N], [C_8_MIM][Tf_2_N]
		[CNC_3_MIM]	1-(3-Cyanopropyl)-3-methylimidazolium	[CNC_3_MIM][Tf_2_N]
		[BMP]	1-Butyl-1-methylpyrrolidinium	[BMP][Tf_2_N]
		[C_6_MPY]	1-Hexyl-4-methylpyridinium	[C_6_MPY][Tf_2_N]
		[ClPr]	Chlorpromazine hydrochloride	[ClPr][Tf_2_N]
[R_4_P]	Bis(2,4,4-trimethylpentyl) phosphinate	Cyphos 104	Trihexyl(tetradecyl)phosphonium	Cyphos IL 104
[TDI]	2,4-Diisocyanate	[BIM]	1-Benzylimidazole	[BIM][TDI]
[TOS]	Tosylate	[C_4_MIM]		[C_4_MIM][TOS]
[TS]	Thiosalicylate	[A336], [PR4]		[A336][TS], [PR4][TS]

**Table 2:** Determination of heavy metal ions in real water samples using IL.

Ions	Sample	Used ILs	Analysis condition	Operation method	Added (*μ*g/L)	Found (*μ*g/L)	Recovery (%)	Type of interference ions	Ref.
Co^2+^	Tap, lake, and rain water (5.0 mL)	25.0 mg of [HMIM][BF_4_] with 80.0 mg of NaPF_6_	R.T.; FAAS	ISFME	0.0–50.0	0.0–71.1	94.1–101.4	Na^+^, K^+^, Ca^2+^, Mg^2+^, Ba^2+^, Fe^2+^, Fe^3+^, Mn^2+^, Cd^2+^, Ni^2+^, Zn^2+^, Pb^2+^, NO_3_^−^, Cl^−^, SO_4_^2−^, Br^−^, and Cu^2+^	[41]

Co^2+^	Mineral, tap, and river water (10.0 mL)	64.0 mg of [HMIM][PF_6_] with 5.0 mg of [HMIM][Tf_2_N]	35°C; FAAS	DLLME	0.0–100.0	0.0–100.9	98.0–102.5	Ag^+^, Hg^2+^, Na^+^, K^+^, Mg^2+^, NH_4_^+^, Ca^2+^, Ni^2+^, Mn^2+^, Cu^2+^, Zn^2+^, Cd^2+^, Fe^3+^, Al^3+^, Rh^2+^, Sn^2+^, Pb^2+^, Cr^3+^, HPO_4_^2−^, CO_3_^2−^, NO_3_^−^, H_2_PO_4_^−^, F^−^, SO_4_^2−^, and PO_4_^3−^	[44]

Cd^2+^	Lake and waste water (15.0 mL)	100.0 *μ*L of [BMIM][PF_6_] with 0.01% PAN	R.T.; FAAS	DLLME	0.0–20.0 × 10^6^	13.2–37.1 × 10^6^	97.6–101.0	Fe^3+^, Zn^2+^, Pb^2+^, Na^+^, K^+^, Ca^2+^ and Mg^2+^	[62]

Cr^4+^	Mineral, sea, and river water (10.0 mL)	150.0 *μ*L of [BMIM][BF_4_] and 0.3 mL of DPC	R.T.; FAAS	LLME	0.0–10.0	0.0–13.2	97.6–99.2	Li^+^, Na^+^, Ca^2+^, Mg^2+^, Ba^2+^, Ag^+^, Mn^2+^, Zn^2+^, Co^2+^, Cu^2+^, Ni^2+^, Cd^2+^, Bi3+, Al^3+^, Pb^2+^, Fe^3+^, and Hg^2+^	[65]
Pb^2+^	Ground and surface water (10.0 mL)	125.0 *μ*L of [BMIM][PF_6_] with 1.0 mL of dithizone	50°C; FAAS	UA-ILDME	0.0–4.0	1.7–5.7	97.3–99.3	Na^+^, K^+^, Ca^2+^, Mg^2+^, Al^3+^, Fe^3+^, Mn^2+^, Co^2+^, Ni^2+^, Zn^2+^, Cr^3+^, and Cd^2+^	[66]

Ag^+^	Photographic waste, mineral, and lake water (10.0 mL)	60.0 *μ*L of [HMIM][BF_4_] with 7.5 × 10^−6^ mol/L of TMK	0°C; UV-Vis	M-CIAME	0.0–50.0	0.0–107.3	98.4–104.8	Li^+^, Na^+^, Pb^2+^, Cd^2+^, Al^3+^, Ba^2+^, Ca^2+^, Co^2+^, Zn^2+^, Mg^2+^, Pd^2+^, Cu^2+^, Ni^2+^, Cr^3+^, Mn^2+^, Cr^4+^, Bi^3+^, Fe^3+^, and Hg^2+^	[67]

Al^3+^, Ga^3+^, and In^3+^	Tap, mineral, and well water (10.0 mL)	75.0 *μ*L of [HMIM][PF_6_] with 1.0 × 10^−5^ mol/L of quinalizarine	R.T.; UV-Vis	UA-ILDME	0.0–50.0	6.7–54.3	86.0–120.5	Li^+^, Na^+^, K^+^, Cl^−^, NO_3_^−^, Ca^2+^, Mg^2+^, Ba^2+^, SO_4_^2−^, Pb^2+^, Ag^+^, Hg^2+^, Co^2+^, Zn^2+^, Cr^3+^, Mn^2+^, Cd^2+^, Ni^2+^, Fe^2+^, and Cu^2+^	[68]

Co^2+^ and Ni^2+^	Tap and mineral water (10.0 mL)	65.0 mg of [HMIM][Tf_2_N] with 1.0 × 10^−3^ mol/L PAN	R.T.; UV-Vis	DLLME	0.0–10.0	0.0–14.5	94.0–102.0	Na^+^, K^+^, Ag^+^, F^−^, Cl^−^, Br^−^, NO_3_^−^, SO_4_^2−^, PO_4_^3−^, SCN^−^, CH_3_COO^−^, Mg^2+^, Ca^2+^, Ba^2+^, Pb^2+^, Cr^3+^, Cr^3+^, Al^3+^, Sb^3+^, Cu^2+^, Cd^2+^, Pd^2+^, and Fe^2+^	[69]

Cd^2+^ and Pb^2+^	Tap, river, and well water (15.0 mL)	120.0 *μ*L of [BMIM][PF_6_], [HMIM][PF_6_] and [OMIM][PF_6_] with 0.4 mg of APDC	R.T.; FAAS	UA-MR- il-DLLME	0.0–15.0	0.0–18.9	97.1–101.6	Na^+^, K^+^, Ca^2+^, Mg^2+^, Mn^2+^, Cu^2+^, Zn^2+^, Cl^−^, and SO_4_^2−^	[70]

Ni^2+^, Co^2+^, Cd^2+^, Cu^2+^, and Pb^2+^	River and lake water (15.0 mL)	Cyphos IL 104	R.T.; LC	IL-UADLLME-SAP	0.2–62.4	0.3–15.3	97.0–102.0	—	[71]

Ag^+^, As^3+^, Cd^2+^, Cr^3+^, Cu^2+^, Hg^+^, Ni^2+^, Pb^2+^, Pt^4+^, Sn^2+^, and Zn^2+^	Communal and industrial wastewater (20.0 mL)	120.0 *μ*L of 8 ILs	20°C; LC-MS	LLME	(Extraction efficiency: 5.0–100.0)			—	[72]

R.T.: room temperature; FAAS : flame atomic absorption spectrometer; LC-MS : liquid chromatography-mass spectrometry.

**Table 3:** Determination of organic pollutants in real samples by IL solutions.

Pollutants	Samples	Used IL	Analysis condition	Operation method	Added (*μ*g/L)	Found (*μ*g/L)	Recovery (%)	LOD (*μ*g/L)	RSD (%)	Ref.
o,p'-DDT, p,p'-DDT, p,p'-DDD, and p,p'-DDE	Snow, rain, lake, and tap water (5.0 mL)	300.0 *μ*L of [BMIM][PF_6_], [HMIM][PF_6_], [OMIM][PF_6_], [BMIM][BF_4_], [EMIM][BF_4_], and [BMIM]NO_3_	R.T.; HPLC	DLLME	5.0	—	85.7–106.8	0.2–0.5	6.0–8.5	[24]

17-*β*-Estradiol-benzoate, 17-*α*-estradiol, and quinestrol	Lake, well, tap, and river water (160.0 mL)	80.0 *μ*L of [BMIM][BF_4_] with 0.3 g of [NH_4_][PF_6_]	R.T.; HPLC	DLLME	0.2–0.9	—	95.5–114.6	0.04–0.05	4.2–8.0	[26]

Phenol, CP, DCP, TCP, and PCP	Aqueous emulsion (10.0 mL)	0.02% (v/v) of [BMIM][PF_6_] in TBP	R.T.; UV-vis	Liquid membranes	Extraction efficiency: 96.9%–99.5%					[79]
		0.2% (v/v) of aliquat 336 in TBP			Extraction efficiency: 90.1%–99.3%					[80]

Phenol, o-cresol, 2-chlorophenol, and resorcinol	Aqueous solution	5.0 mg/mL of [BMIM][Tf_2_N], [HMIM][Tf_2_N], and [PMIM][Tf_2_N]	33°C; color reaction	Liquid extraction	—	—	99.99	—	—	[81]

Gallic acid, vanillic acid, and vsyringic acid	Salty aqueous solution (20.0 mL)	5 wt% of [BMIM](SCN), [BMIM][TOS], [BMIM][N(CN)_2_], [BMIM][CH_3_CO_2_], [BMIM]Cl, [BMPIP]Cl, and [BMPYR]Cl	R.T.; UV-vis	ATP	Extraction efficiency: 66.0%–94.6%				—	[82]

NT, DNT, and TNT	Tap and lake water (5.0 mL)	26.0 mg of [HMIM][Tf_2_N] and [C_6_MPY][Tf_2_N]	R.T.; HPLC	DLLME	5.0–20.0	4.7–20.0	93.2–101.0	0.7–1.1	3.1–4.3	[83]

BPA, 4-CP, t-BP, OP, t-OP, and NP	Deionized and sea water (20.0 mL)	10.0 *μ*L of [BMIM][Tf_2_N]	R.T.; HPLC	DLLME	—	—	67.6–114.0	0.8–4.8	2.8–11.0	[84]

BPA and 2-naphthol	Tap, lake, and river water (5.0 mL)	35.0 *μ*L of [OMIM][PF_6_]	R.T.; HPLC	CIA-ME	20.0	—	97.1–108.1	0.6–0.9	2.3–4.1	[85]

BPA	Tap, reservoir, and beach water (0.05 mL)	5.0 *μ*L of TOAP	20°C; HPLC	ATPM	100.0–1500.0	95.5–1559.0	95.5–109.9	4.3–4.6	2.9–4.5	[86]

TCL, DOX, BPA, TCS, and MTCS	Sea, WRTR, and tap water (5.0 mL)	115.0 *μ*L of [BMIM][PF_6_], [HMIM][PF_6_], and [OMIM][PF_6_]	R.T.; HPLC	SILM-DS	8.0–50.0	6.3–47.7	74.5–106.9	0.1–0.8	2.2–6.5	[87]

**Table 4:** Preparation methods of IL-modified sorbents.

Substrate	Used IL	Preparation process	Method	Ref.
Graphene, CNT	(BMP)[Tf_2_N]	Impregnation	57.0% graphite powder, 14.0% (BMP)[Tf_2_N], 10.0% MWCNTs and 13.0% macrocyclic ligand, and 6.0% paraffin oil (weight ratio)	[94]
Activated carbon	TOMAS		0.18 g of PSAC and 0.12 g of TOMAS	[95]
Three resins	Cyphos IL 101		0.5 g of Cyphos IL 101, 1.0 g of amberlite XAD-4, XAD-16, and XAD-1180, mixing in 5.0 mL of ethanol for 6 h	[96]
XAD-4	(BMP)[Tf_2_N]		1.0 g of XAD-4 and 100.0 *μ*L of (BMP)[Tf_2_N], mixing in 100.0 mL acetone for 12 h at RT	[100]
Silica	[NH_2_EBIM][PF_6_]		3.0 mL of [NH_2_EBIM][PF_6_] and 10.0 g of silica, mixing in methanol for 2.5 h	[108]
Silica	[EMIM][Tf_2_N], [OMIM][Tf_2_N]		10.0 g of Sil-NH_2_, 5.0 mL of [EMIM][Tf_2_N], or 7.2 g of [OMIM][Tf_2_N], mixing in toluene at room temperature (RT)	[110]
Graphene, CNT	[BMIM][PF_6_]		20.0% [BMIM][PF_6_], 18.0% ionophore, 49.0% graphite powder, 10.0% MWCNT, and 3.0% nanosilica (weight ratio)	[116]
Graphene oxide	[BMIM][PF_6_]		500.0 *μ*L of [BMIM][PF_6_] and 15.0 mg of MGO, mixing for 30.0 min	[117]
Graphene oxide	[HMIM]Gly		0.1 g of IL and 0.1 g of Fe-GO, mixing in 10 mL of methyl alcohol for 2 h	[118]
MOF	[BMIM]Cl		0.5 g of [BMIM]Cl and 10.0 mL of MIL-100(Fe), mixing in aqueous solution for 15 h at RT	[126]

Silica	[ClPr][Tf_2_N]	Chemical modification	2.0 g of [ClPr][Tf_2_N], 6.0 g of silica, 5.0 ml of tetraethoxysilane, and 1.0 ml of 25.0% aqueous ammonia solution, mixing in 60.0 mL of toluene at 25°C for 5 h	[105]
Silica	[CNC_3_MIM][Tf_2_N]		20.0 g of silica and 20.0 mL of 3-aminopropyltrimethoxysilane, reacting in 150.0 mL of toluene	[119]
			5.0 g of the aminated silica and 3.0 g of [CNC_3_MIM][Tf_2_N], reacting in 100.0 mL of toluene	
*β*-CD	[BIM][TDI]		First, *β*-CD (10.0 mmol) and Ts_2_O (15.0 mmol) mixing in 250.0 mL of water at RT for 2 h. Second, *β*-CDOT (78.0 mmol) and BIM (7.8 mmol) reacting at 90°C	[127]
			Third, 1.0 g of *β*CD-BIMOT stirred with TDI (10.0 mol) at 70°C for 24 h	
Silica	[BMIM]Br		400.0 mg of silica and 462.3 mg of [C4MIM]Br, reacting in 50.0 mL methanol at 30°C for 4 h	[128]
			1.9 mL of ethylene dimethacrylate and 10.0 mg of azobisisobutyronitrile were added and shaked at 60°C for 24 h	
MIPIL	3-(Anthracen-9-ylmethyl)-1-vinyl-1h-imidazol-3-ium chloride		1-vinylimidazole (0.94 g) and 9-chloromethylanthracene (1.133 g), reacting in acetonitrile (30 mL) at 70°C for 12 h. Then, the obtained IL monomer (64 mg), EGDMA (198 mg), p-NA (7 mg), and AIBN reacting at 60°C for 12 h	[129]
MIP	[VBIM]Cl		CS (1.5 mmol), [VBIM]Cl (3.6 mmol), EDMA (8.0 mmol), and AIBN (20.0 mg), reacting in 2.5 mL N,N-dimethylformamide and 4.0 mL toluene and 1.0 mL methanol at 60°C for 24 h	[130]

**Table 5:** Adsorption of pollutants in real water samples by IL-modified sorbents.

Pollution	Source of water samples	Analytical method	Added (*μ*g/L)	Found (*μ*g/L)	Recovery (%)	LOD (*μ*g/L)	RSD (%)	Ref.
Hg^2+^	Sea and waste	Potentiometer	0.0–0.08	0.02–0.1	98.6–103.2	0.008	2.8	[94]
Cd^2+^	Drinking	Potentiometer	1.4 × 10^7^	—	97.8–104.6	14.4	1.6–3.2	[95]
Hg^2+^	Mineral, tap, and river	SPE	0.0–1.0	0.03–1.1	96.2–103.0	2.3 × 10^−3^	2.7	[105]
Cd^2+^	Tap and lake	SPE	0.0–1.0	0.0–1.2	97.0–104.0	8.9 × 10^−3^	2.3	[108]
Cd^2+^	Tap and waste	Adsorption in vitro	1.0–2.0 × 10^3^	—	97.8–98.8	—	—	[110]
Pb^2+^	Tap, river, and waste	Potentiometer	26.7–1.3 × 10^4^	24.9–1.6 × 10^4^	95.0–102.0	0.4	<1.0	[116]
Cu^2+^, Co^2+^, Cr^2+^, Ni^2+^, Zn^2+^, and Cd^2+^	Waste, river, and mineral	Adsorption in vitro	20.0	19.1–91.0	90.5–107.5	0.1–1.0	<3.2	[117]
Al^3+^, Cr^3+^, Cu^2+^, and Pb^2+^	Lake and waste	Adsorption in vitro	0.0–100.0	0.0–170.9	88.4–117.8	0.5–30.0 × 10^−3^	1.4–6.0	[118]
Pb^2+^ and Cd^2+^	Tap, domestic, and industrial	Adsorption	2.0–12.0 × 10^3^	1.1–19.2 × 10^3^	97.4–99.8	—	—	[119]
DBP	River and canal	Adsorption in vitro	1.0–10.0	—	80.0–87.0	0.15	4.3–7.8	[96]
4-CP	Drinking, ground, lake, sea, and waste	Adsorption in vitro	25.0–75.0 × 10^3^	0.5–8.5 × 10^3^	88.6–98.1	9.8 × 10^3^	—	[100]
12 PAHs	Mineral, river, and sea	SPE	2.0–20.0	1.9–20.1	97.0–103.5	2.0–5.5 × 10^−3^	3.0–4.9	[126]
6 phenols	Tap and river	SPE	0.5–1.0 × 10^3^	—	87.0–116.3	0.2–0.4	1.0–3.4	[127]
2,4-DCP, BPA, and 2,4-DNP	Industrial, dyeing, textile, river, and plant effluent	SPE	6.0–500.0	—	71.1–115.7	—	1.1–11.3	[128]
*p*-NA	River, tap, and lake	Adsorption in vitro	5.0–45.0 × 10^3^	—	89.0–114.0	—	—	[129]
CS	Surface	SPE	1.0–5.0	—	81.0–110.1	1.0 × 10^−3^	1.2–7.6	[130]
